# Umbilical and Placental Derivatives in Temporomandibular Joint Treatment: A Systematic Review

**DOI:** 10.3390/jcm13237002

**Published:** 2024-11-21

**Authors:** Karolina Lubecka, Maciej Chęciński, Kamila Chęcińska, Filip Bliźniak, Tomasz Wach, Mariusz Szuta, Dariusz Chlubek, Maciej Sikora

**Affiliations:** 1 Department of Oral Surgery, Preventive Medicine Center, Komorowskiego 12, 30-106 Kraków, Poland; lubeckarolina@gmail.com (K.L.); maciej@checinscy.pl (M.C.); fblizniak@gmail.com (F.B.); 2 National Medical Institute of the Ministry of Interior and Administration, Wołoska 137, 02-507 Warsaw, Poland; sikora-maciej@wp.pl; 3 Department of Maxillofacial Surgery, Hospital of the Ministry of Interior, Wojska Polskiego 51, 25-375 Kielce, Poland; 4 Department of Glass Technology and Amorphous Coatings, Faculty of Materials Science and Ceramics, AGH University of Krakow, Mickiewicza 30, 30-059 Kraków, Poland; checinska@agh.edu.pl; 5 Department of Maxillofacial Surgery, Medical University of Lodz, Żeromskiego 113, 90-549 Lodz, Poland; tomasz.wach@umed.lodz.pl; 6 Department of Oral Surgery, Jagiellonian University Medical College, Montelupich 4, 31-155 Kraków, Poland; m.szuta@wp.pl; 7 Department of Biochemistry and Medical Chemistry, Pomeranian Medical University, Powstańców Wielkopolskich 72, 70-111 Szczecin, Poland

**Keywords:** temporomandibular joint, temporomandibular disorders, umbilical cord, placenta, allografts, amniotic membrane, mesenchymal cells

## Abstract

**Objectives**: This review aimed to gather and summarize the existing information on the clinical application of allogeneic umbilical and placental derivatives in the treatment of temporomandibular joint disorders. **Methods**: Research on the impact of the use of umbilical and placental derivatives on reducing pain and improving mobility in the temporomandibular joint was included in the article. Medical databases, including ACM, BASE, Cochrane, Scopus, Google Scholar, ClinicalTrials.gov, and PubMed, were searched. The final search was conducted on 20 October 2024. **Results**: Out of the 43 records found, 5 were considered eligible for further analysis and showed that the use of placental and umbilical derivatives has the greatest potential in the treatment of ankylosis. The intra-articular administration of these tissues into the TMJ brings beneficial results, but they are similar to other, parallel methods, such as PRP or corticosteroids. **Conclusions**: The studies discussed may guide researchers in expanding clinical trials, particularly by including more patients with TMDs, and have promising potential in ankylotic disorders, where amniotic membrane use has shown clear benefits.

## 1. Introduction

### 1.1. Background

Temporomandibular disorders (TMDs) affect the temporomandibular joint (TMJ) and the muscles controlling mandible movement, causing pain, stiffness, or dysfunction. TMDs can result from injuries, arthritis, stress, or habits like teeth grinding, leading to difficulty in chewing, speaking, or opening the mouth fully [1]. Wilkes’ classification divides the advancement of TMDs into five stages. The first manifests itself only by reducing disc displacement. There are no symptoms such as pain or mobility restriction. In the second stage, an occasional pain occurs. Stages 3, 4, and 5 involve non-reducing disc displacement. In Stage 3, frequent headaches and arthralgia episodes coexist with painful mastication. The range of motion in the joint is limited and locking is present. In Stage 4, functional disturbance increases, and non-reducing disk displacement is chronic along with headaches and articular pain. There are also radiologic signs of degenerative changes. In Stage 5, pain is variable and co-occurs with osteoarthritis, crepitus, grinding symptoms, and restricted motion [2].

The latest classification, the International Classification of Orofacial Pain (ICOP) of 2020, divides temporomandibular joint pain into numerous types, attributed to arthritis, disc displacement, degenerative joint disease, or subluxation [3]. TMJ pain results from inflammation due to trauma, wear, autoimmune disease, or infection. Pain may be one of the symptoms of osteoarthritis; however, osteoarthritis may also occur painlessly. TMJ pain attributed to disc displacement occurs due to mechanical conflict in articular structures. TMJ pain attributed to subluxation is an acute pain occurring while tissues are being overstretched [3].

Following the ICOP 2020, the degenerative TMJ disease diagnostic criteria include (1) arthralgia, (2) acoustic symptoms in the last 30 days or during the examination, and (3) crepitus detected with palpation during maximal unassisted or assisted mouth opening, lateral movement, or protrusion. Severe stages of degeneration are characterized by difficult-to-control pain [3]. Symptomatic pain relief methods include systemic pharmacotherapy, physiotherapy, splint therapy, and minimally invasive surgery [4,5]. The latter includes TMJ rinsing and intra-articular injections [6,7,8]. Causal treatment of advanced degenerative joint disease is difficult and dichotomous. The first direction involves attempts to regenerate the bone tissue and cartilage of the TMJ. For this purpose, blood-derived autografts and mesenchymal stem cells from adipose tissue are used [9]. Another option is to replace the degenerated structures of the temporomandibular joint. An extreme option here is a total arthroplasty and replacing the joint with an endoprosthesis, benefiting quality of life [10].

Another indication for replacing the TMJ with an artificial equivalent is losing the ability to move the joint surfaces relative to each other due to their fusion. This situation is called ankylosis [11]. It results primarily from post-traumatic TMJ immobilization. Simply put, the body treats the temporal bone and the head of the mandible (or its stump) surfaces as single bone fragments, creating a fusion between them [12]. The arthroplasty procedure does not always involve removing and replacing all TMJ tissues with alloplastic material. Depending on the severity of the ankylosis and whether it is fibrous or bony, it is often possible to remove the adhesion itself, while sparing the surrounding tissues. An important therapeutic challenge with prognostic significance is the separation of newly formed joint surfaces to prevent re-union [13,14].

### 1.2. Rationale

**Figure 1:**
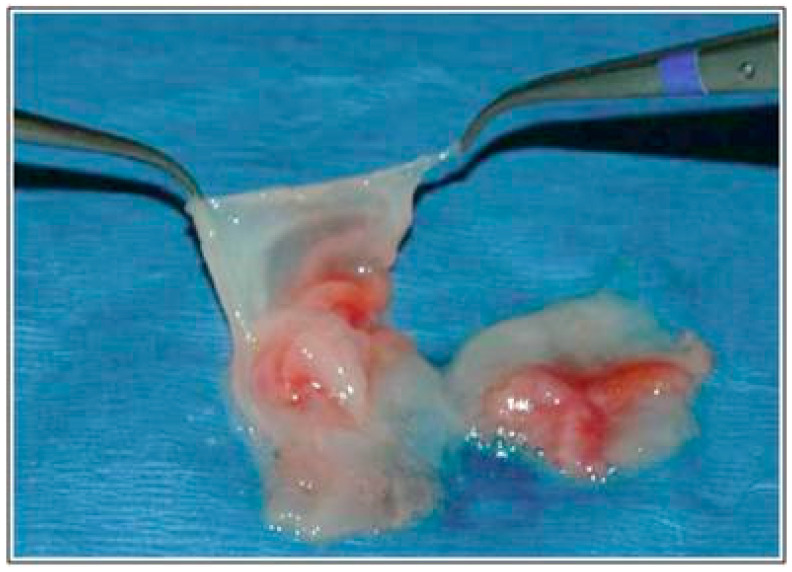
Dissection of the umbilical cord. Author: Johnlancer123. License: CC BY-SA 3.0 (https://creativecommons.org/licenses/by-sa/3.0; accessed on 18 October 2024).

TMDs can be treated with biological methods, such as injections of blood products or stem cell transplantation [9]. Mesenchymal stem cells (MSCs) are widely used in regenerative medicine due to their immunomodulatory and multipotential properties. Apart from bone marrow or adipose tissue, MSCs can be obtained from human umbilical cord tissue (Figure 1). This source provides harmless cell retrieval, low immunogenicity, and abundance in tissues [15]. When administered into the joint, the cells’ products show a strong anti-inflammatory effect, comparable to dexamethasone [15]. MSCs can be used both in live form and as a lysate, which is equally effective [16].

**Figure 2:**
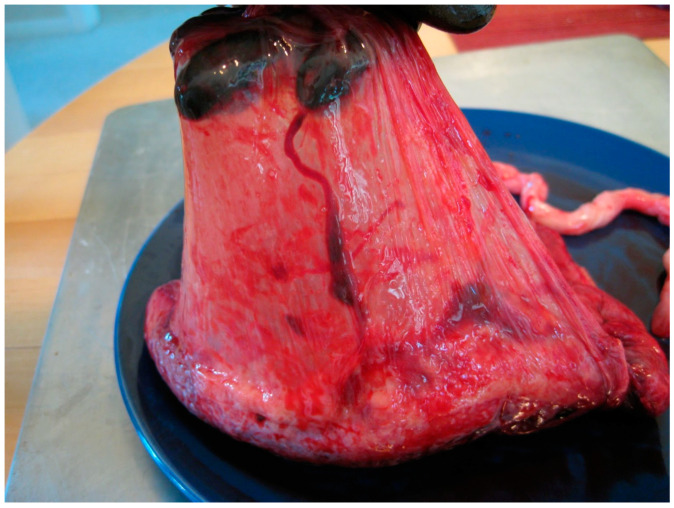
Amniotic sac. Cropped. Author: moppet65535. License: CC BY-SA 2.0 (https://creativecommons.org/licenses/by-sa/2.0; accessed on 18 October 2024).

The human amniotic membrane (HAM) is also a source of MSCs (Figure 2). So far, it has been used in extensive, non-healing wounds, burns, and ulcers treatment. According to Odet et al. [17], HAM is also used to treat medication-related osteonecrosis of the jaw due to its high regenerative potential. The benefits of the above therapies led to an attempt to use them in the surgical treatment of TMJ ankylosis, as they promote the regeneration of proper fibrous cartilage. Studies show that using HAM to treat ankylosis reduces the chance of re-ankylosis occurrence in the future [18].

It was found that joint replacement enhances the quality of life of patients with advanced TMD [10]. This justifies the search for invasiveness reduction, provided that similar improvements in life quality, articular pain, mandibular mobility range, and mastication efficiency are maintained. There are emerging TMD treatment strategies involving the use of autologous MSCs [9]. When properly stimulated, they can differentiate into various cell types, including chondrocytes, regenerating cartilage tissue. The satisfactory effects of using autologous fat-derived MSCs in the treatment of TMDs open the possibility of using allogeneic ones from the umbilical cord and placenta, which is a unique direction in the search for methods of treating TMJ internal derangement, degenerative joint disease, trauma, and ankylosis.

So far, various studies, both in vitro and in vivo, have proven the significant anti-inflammatory properties of amniotic MSCs. These consist of, among others, inhibiting the proliferation of T and B lymphocytes and inhibiting pro-inflammatory dendritic cells, natural killers, or macrophages [19,20], which results in the significant effectiveness of this type of treatment. These findings suggest MSCs could be useful for treating T cell-mediated diseases, such as transplant rejection and autoimmune disorders, but further studies are needed to evaluate their long-term survival and effectiveness in vivo.

On the other hand, amniotic cells can also stimulate the host’s inflammatory response and induce the proliferation of regulatory T lymphocytes in vitro. They are capable of constitutively expressing human leukocyte antigen (HLA), which is one of the main factors responsible for the proliferation of the aforementioned lymphocytes. The immunogenicity of MSCs in some cases may appear in the form of local rejection of the graft or a systemic reaction—graft-versus-host disease (GvHD) [19,20,21].

### 1.3. Objective

This review was conducted to identify, synthesize, and discuss the evidence on the clinical use of allogeneic umbilical cord and placental derivatives in TMD treatment.

## 2. Materials and Methods

The systematic review was prepared under the Preferred Reporting Items for Systematic Reviews and Meta-Analyses (PRISMA) guidelines and reported following the PRISMA Checklist [22].

### 2.1. Protocol and Registration

The protocol for this review has not previously been published as a separate journal article. It has been registered on the Open Science Framework (OSF). OSF registration number: osf.io/9mnjz.

### 2.2. Eligibility Criteria

Eligibility criteria were determined using the SPIDER method [23]. Detailed criteria for each domain are tabulated (Table 1).

### 2.3. Information Sources

Medical databases were searched using the Association for Computing Machinery Guide to Computing Literature (ACM), Bielefeld Academic Search Engine (BASE), Cochrane Central Register of Controlled Trials (Cochrane), Elsevier Scopus (Scopus), Google Scholar with the “allintitle”: command (Scholar), National Library of Medicine Clinical Trials (ClinicalTrials.gov), and National Library of Medicine Pubmed (Pubmed) engines. A gray literature search using the Google engine and a manual reference search were also performed (Gray).

### 2.4. Search Strategy

In the first stage, multiple preliminary searches were conducted to identify subsequent keywords and expand the query. The ultimate query was as follows: “(temporomandibular OR tmj) AND (umbilical OR “Wharton’s jelly” OR placenta OR placental OR amnion OR amniotic) AND (allogenic OR allogeneic OR allograft OR allografts OR graft OR grafts OR lysate OR lysates) AND (clinical OR trial OR human OR patient OR patients OR case OR cases)”. All final searches were conducted on 20 October 2024. To ensure the comprehensiveness of searches, no search engine filters were applied.

### 2.5. Selection Process

The records identified after the search in scientific databases were entered into The Rayyan automation tool (version 2024-10-20, Qatar Computing Research Institute, Doha, Qatar and Rayyan Systems, Cambridge, MA, USA). Then, manual deduplication was performed (M.C.). After that, two independent researchers (K.L. and F.B.) performed a blinded screening of the records based on titles and abstracts. In the case of disagreement between the reviews, the records were qualified for the next stage. Then, Cohen’s kappa value was assessed using MedCalc (version 23.0.6; MedCalc Software Ltd., Ostend, Belgium). In the next stage, a full-text evaluation of the papers was performed (K.L. and F.B.), and, if necessary, a third reviewer (M.C.) was asked for a decisive vote.

### 2.6. Data Collection Process

Two independent researchers (K.L. and F.B.) extracted data. In case of discrepancies, the third researcher (M.C.) decided. Data were collected without using any automation tools. Researchers entered data into tables to present study characteristics and results. The discussion addressed the entire content of the research reports, not just the information presented in the results section of this paper. The numerical data were presented in tabular form using the Google Workspace package (version 2024.10.18; Google LLC, Mountain View, CA, USA).

### 2.7. Data Items

For characterization, the following data items were collected: (1) first author of the research report, (2) year of publication of the paper, (3) size of the study sample, (4) size of the control, (5) diagnosis, and (6) intervention in the study sample. The initial and subsequent values recorded during the observations were taken as outcome data items. Regardless of the original scale, quality of life was converted into a percentage. Articular pain intensity was converted to values from 0 to 10, irrespective of whether they were from a visual analog, numerical rating, or any other scale. The range of maximum unassisted abduction expressed in millimeters was preferred as a determinant of the range of jaw mobility. If this variable was unavailable, any other variable defining the range of mouth opening or, failing this, any characteristic of mandibular mobility was extracted.

### 2.8. Study Risk of Bias Assessment

The risk of bias was determined using the Cochrane tool “Risk Of Bias In Non-randomized Studies—of Interventions” (ROBINS-I). The evaluation was performed by two independent authors (K.L. and F.B.); if necessary, a third author (M.C.) decided. The risk of bias in study assessments was presented in tabular and graphical form using the Robvis tool (version 2023, University of Bristol, Bristol, UK).

### 2.9. Effect Measures

To investigate the effectiveness of using umbilical and placental derivatives in TMD treatment, the mean difference and 95% confidence interval were calculated where possible. The MedCalc Software (version 23.0.6; MedCalc Software Ltd., Ostend, Belgium) was used for this purpose.

### 2.10. Synthesis Methods

The syntheses were carried out both tabularly and descriptively, with chart visualization. The Google Workspace package was used (version 2024.06.28; Google LLC, Mountain View, CA, USA). Mean differences were calculated using the MedCalc program (version 22.030; MedCalc Software Ltd., Ostend, Belgium).

### 2.11. Certainty Assessment

The investigators assessed the quality of evidence for each eligible outcome. The following were considered: (1) the number of source reports, (2) the risk of bias scores, (3) the size of the patient groups included in the studies, and (4) the mean difference between pre- and post-procedure values.

## 3. Results

### 3.1. Study Selection

**Figure 3:**
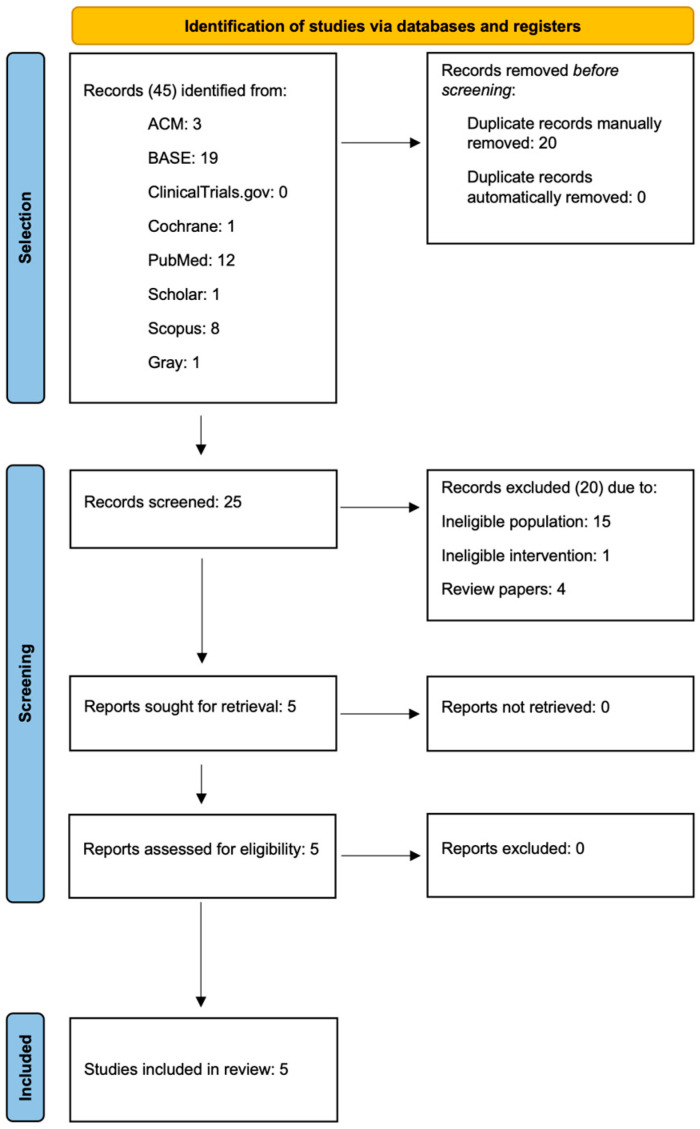
PRISMA flow diagram.

Independent searches using all search engines identified above identified a total of 45 records (Figure 3). After manual deduplication, a screening of 25 remaining records was performed, of which 5 were ultimately found to be eligible. There were no discrepancies between researchers during the selection process.

### 3.2. Study Characteristics

The study groups did not exceed 13 patients and constituted a total sample of 29 subjects (Table 2). There was no control sample in any of the studies. The diagnoses and interventions were heterogeneous.

### 3.3. Risk of Bias in Studies

**Figure 4:**
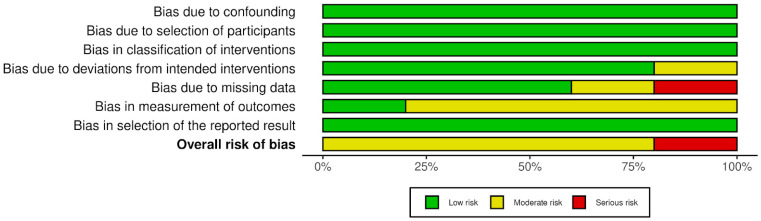
Risk of bias in studies—part 1.

**Figure 5:**
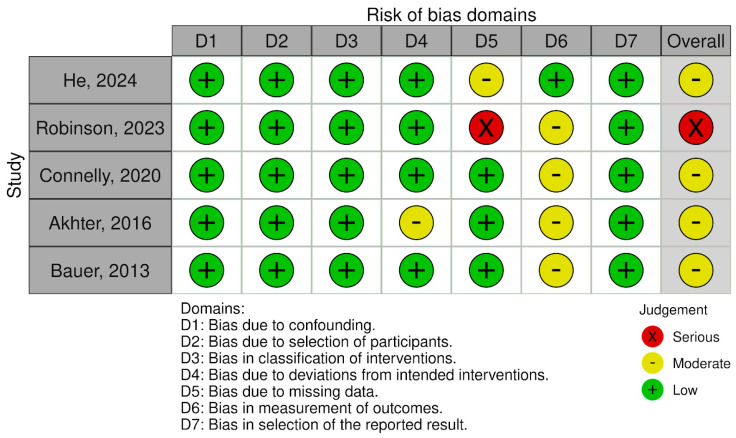
Risk of bias in studies—part 2 [24,25,26,27,28].

In each study, except He et al., it cannot be ruled out that the researchers carrying out the measurements knew the interventions received by the patients. Due to this concern, these the studies were rated as having a moderate risk of bias. Assessment details are presented in Figure 4 and Figure 5.

### 3.4. Results of Individual Studies

The study by He et al. is a case report of a 27-year-old male patient who presented with limited jaw opening and a history of trauma that occurred as a result of a traffic accident 7 years earlier [24]. Fractures involved the midface and bilateral condyles of the mandible. The midfacial fractures were fixed with resorbable bone plates, while the condylar fractures were treated conservatively, resulting in limited mandibular mobility afterward. Radiological examinations, including cone beam computed tomography, revealed bilateral bony ankylosis of the TMJs. Therefore, it was decided to perform arthroplasty with interpositional umbilical fat grafting. In post-operative examinations, the maximum opening of the mandible increased to a physiological value of 40 mm, which can be considered a success of this therapeutic method.

In the Robinson et al. study, patients with TMD caused by articular disc degeneration were administered an intra-articular injection of Wharton’s jelly tissue allograft [25]. The injection was preceded by palpation of the area, allowing the provider to insert the needle accurately. Patients were followed up at 30, 60, and 90 days after the procedure, where computed tomography images, range of mandibular motion, and pain were assessed. On final examination at 90 days after the procedure, pain had decreased in all patients by 50–100%. The extent of improvement in mouth opening varied among patients and ranged from 3 to 6 mm achieved with treatment.

The purpose of Connelly et al.’s research was to evaluate the impact of implanting a cryopreserved viable osteochondral allograft combined with a viable cryopreserved umbilical cord tissue allograft into the TMJs of patients who had undergone discectomy and were suffering from degenerative joint disease [26]. The tissue was placed in the joint cavity during an open surgery. The patients were then evaluated, on average, 15 months after the procedure (the follow-up time varied between patients). During follow-up examinations of patients, researchers noted pain relief, increased jaw mobility, and improved quality of life, which encourages further development of this treatment method in the future.

Akhter et al. aimed to prove that an amniotic membrane can be a sufficient and suitable material to separate fragments in TMJ ankylosis and to prevent re-ankylosis [27]. They pointed out that it is an economical and biocompatible option. The authors of the study selected 13 patients (10–35 years old) with a unilateral or bilateral ankylosis confirmed clinically and radiologically. During each surgical procedure, an amniotic cap containing 10–15 layers of a sterilized amniotic membrane obtained from a tissue bank was placed over the condylar head. The results of the conducted study are satisfactory and the amniotic membrane, indeed, was evaluated as a promising alternative to existing and used materials.

The study by Bauer et al. is a case report of a 53-year-old woman with a giant cell tumor located in the left condylar process [28]. The neoplasm involved also the entire TMJ cavity; therefore, a decision was made to perform a radical resection including the condylar process and the articular disc. During the same surgery, the removed joint tissues were reconstructed using a costochondral graft combined with an allogenic human amniotic membrane (HAM). The post-operative wound healed properly and there were no complications in the process of recovery. Ankylosis did not occur at the follow-up visits, as the patient presented with a 32 mm interincisal opening 8 months after surgery.

### 3.5. Result of Syntheses

A graphical representation of the results is shown in Table 3, Table 4, Table 5 and Table 6. Quality of life assessment was only included in Connelly et al. [26]. Robinson et al., Akhter et al., and Bauer et al. did not address this topic in any way, hence its absence in Table 3 and Table 4 [25,27,28]. Information about articular pain was present only in Connelly et al. and Robinson et al. (Table 5) [25,26]. Akther et al. and Bauer et al. did not include such data, hence its absence in the table [27,28]. Table 6 presents the results of the most frequently included variable in the source studies, i.e., mandibular mobility.

### 3.6. Certainty of Evidence

The following variables were examined in the included papers: pain reduction, improvement in jaw mobility, and quality of life (Table 7). Due to the lack of baseline values for interincisal opening in Robinson et al., the percentage values of mean difference and standard deviation could not be calculated [25].

## 4. Discussion

Human umbilical cord and amniotic membrane tissues have been thoroughly investigated in animal models, such as rabbits or mice, in treating inflammatory TMJ diseases [29]. The results of these studies are promising in the context of clinical use. According to Kim et al., who conducted research on rabbits, human umbilical cord MSCs have the ability to differentiate into chondrocytes [15]. This underlines their regenerative and protective potential for cartilage tissue in TMJ-OA treatment. They also have an anti-inflammatory effect, stronger than intra-articular steroids commonly administered in TMJ-OA therapy [30]. After administration, MSCs persist in the tissues for at least 4 weeks, which enhances their beneficial effect [31]. Despite being xenogeneic, human umbilical cord tissues appear to function properly in a rabbit model, which bodes well for allografts [29]. The exact doses of MSCs for specific diseases are not still known and need to be further investigated. In the research conducted by Ward et al., MSCs were tested in a lysate form administered by intra-articular injection [16]. This way of processing cells did not deprive them of beneficial activity, and it also had an anti-inflammatory effect as myeloperoxidase concentration decreased and tissue edema was reduced.

The transplantation of tissues such as muscle grafts and HAM are also effective in the treatment and prevention of temporomandibular joint ankylosis. For the purpose of Tuncel et al.’s study, ankylosis was intentionally induced in rabbits’ temporomandibular joints [18]. One group was treated with gap arthroplasty excising pathological tissue, repositioning anatomical structures, and restoring proper alignment and function. The other group was treated with interpositional arthroplasty, during which previously exposed joint surfaces were covered with a pouch made of HAM for the purpose of their regeneration. Post-operative follow-up examinations showed greater mandibular mobility in rabbits after HAM than after gap arthroplasty. In the post-gap arthroplasty joints, fibrous adhesions were found, whereas after HAM application there were none. This leads to the conclusion that the superiority of HAM application was demonstrated in a rabbit model.

In Akhter et al.’s study, bony TMJ ankylosis was treated with an amniotic membrane, which was applied to bone fragments after condylectomy [27]. This resulted in an increase in the interincisal opening from 0–15 mm to 32–35 mm, which is an increase of over 450%. According to Bauer et al.’s study, the use of the amniotic membrane has also been proven effective in the reconstruction of the TMJ after extensive resection due to a neoplasm [28]. The patient showed an improvement in maximum mouth opening by 113% from the pre-operative state. The choice of this material resulted in the absence of ankylosis in the post-operative period.

Comparing the improvement in parameters after using placental and umbilical derivatives in relation to other TMD treatment methods shows promising results. Post-operative TMJ joint pain was reduced by 75% in the Robinson et al. study, where Wharton’s jelly tissue allograft was injected into the affected TMJ [25]. This is a similar value to that after platelet-derived growth factor intra-TMJ administration (74.48%) and slightly better than post-dexamethasone outcomes (67.01%), according to Agostini et al. [32].

Using umbilical derivatives brings beneficial effects in the treatment of degenerative joint disease. In Connelly et al.’s study, an osteochondral graft and cryopreserved umbilical cord tissue was implanted as a reconstruction after TMJ discectomy due to degenerative joint disease [26]. During follow-up visits 60 weeks after the procedure, pain reduction and mouth opening range were assessed. Pain decreased from preoperative values by 66.67%, while the range of mandibular abduction increased by 16.12%. In the study by Pihut et al., the intensity of TMJ pain was investigated following the intra-articular administration of platelet-rich plasma [33]. After one week the improvement was 56.92%, and after 6 weeks it was 90.77%, which was significantly greater than in Connelly et al. [26].

Nevertheless, methods involving placental and umbilical derivatives prove to be worth further investigation.

### 4.1. Limitations

The evidence collected during this review is severely limited by the paucity of sources. Extensive searches identified only four reports indexed in medical databases. The fifth report was obtained from gray literature sources. Searches on social media and from references yielded no results. It should therefore be concluded that clinical attempts to use derivatives of the umbilical cord and placenta in TMD treatment are unique. However, these attempts are not a new idea. A report on the first known case was published in 2013, with subsequent papers appearing approximately every three years.

The limited number of studies included in this systematic review displayed considerable heterogeneity. Differences in study designs, methodologies, and outcome measures contributed to variability in findings. Most studies were characterized by small sample sizes and limited statistical power, potentially reducing the generalizability of the conclusions. The lack of control groups further constrained the ability to establish causal relationships and control for confounding factors. These limitations warrant careful consideration in interpreting the findings and their implications for clinical applications and future research.

The query was prepared in English, which excluded the identification of sources not indexed in this language, such as those lacking English keywords or an English version of the title.

### 4.2. Implications

The use of placental and umbilical cord derivatives in the treatment of TMD is promising, but still under-researched. There are relatively few papers on this topic, but the existing ones draw attention in particular to the considerable regenerative properties of this type of tissue. Therefore, it seems worthwhile to address this topic in future clinical trials, as it could develop into a scientifically validated alternative technique to the routinely used surgical methods of TMD treatment, including intra-articular injections and arthroplasty.

### 4.3. Future Perspectives

The studies described and compared above may serve as a guide for researchers who wish to expand the scope of clinical trials in the future. One direction that could be pursued is designing studies that include more patients with unilateral or bilateral TMDs that do not respond to non-surgical treatment. Introducing control groups could strengthen the reliability of future studies. Implementing prospective design, randomization, and double-blinding would help reduce bias. More extensive follow-up periods are also recommended to better assess long-term outcomes. An especially rewarding field seems to be ankylotic disorders, in which the use of the amniotic membrane has shown clear results.

## 5. Conclusions

The use of umbilical and placental derivatives in TMD treatment demonstrates potential for future therapeutic applications. However, few clinical studies have addressed this topic. The application of allogenic human amniotic membrane appears promising in cases of temporomandibular joint ankylosis. Umbilical cord derivative allografts require further investigation regarding mesenchymal stem cells’ potential for long-term cartilage regeneration.

## Figures and Tables

**Table 1:** Eligibility criteria.

	Criteria
Sample	TMD patients excluding cadavers
Phenomenon of Interest	Treatment of TMDs with umbilical cord or placenta derivatives
Design	Any primary clinical study, regardless of the presence of randomization and control, including case series and single cases
Evaluation	Any method of measuring the quality of life, the severity of articular pain, or the range of mandibular mobility
Research type	Quantitative, qualitative, and mixed research

**Table 2:** Study characteristics.

First Author, Publication Year	Sample Size	Diagnosis	Intervention in the Study Sample	Variables Included in the Study
He, 2024 [24]	1	TMJ ankylosis	TMJ arthroplasty with interpositional umbilical fat grafting	Mandibular mobility
Robinson, 2023 [25]	5	Degenerative joint disease	Wharton’s jelly tissue allograft application in the affected TMJ	Articular pain, mandibular mobility
Connelly, 2020 [26]	9	Degenerative joint disease	Interpositional cryopreserved viable osteochondral allograft and viable cryopreserved umbilical cord tissue implantation after TMJ discectomy	Health-related quality of life, articular pain, mandibular mobility
Akhter, 2016 [27]	13	TMJ ankylosis	Application of amniotic membrane over the TMJ condylar process	Mandibular mobility
Bauer, 2013 [28]	1	Giant cell tumor of the condylar process	TMJ reconstruction with amniotic membrane after condylar tumor resection	Mandibular mobility

TMJ—temporomandibular joint.

**Table 3:** Post-operative percentage changes in (1) articular pain—preoperative baseline is 100% and (2) mandibular mobility improvement—preoperative baseline is 100%. TMJ—temporomandibular joint.

First Author, Publication Year	Treatment Method	Articular Pain (Final vs. Baseline)	Mandibular Mobility (Final vs. Baseline)
He, 2024 [24]	TMJ arthroplasty with interpositional umbilical fat grafting	N/A	192%
Robinson, 2023 [25]	Umbilical cord tissue allograft	25%	N/A
Connelly, 2020 [26]	Articular disc replacement with umbilical cord allografts	33%	16%
Akhter, 2016 [27]	Ankylosis correction with human amniotic membrane	N/A	453%
Bauer, 2013 [28]	TMJ reconstruction with human amniotic membrane	N/A	113%

N/A—not applicable.

**Table 4:** Health-related quality of life as a percent.

First Author, Publication Year	Label	Baseline	60 Weeks *
Connelly, 2020 [26]	Mean value	N/S	56.5%
	Standard deviation	N/S	23.0%

*—mean follow-up time; N/S—not specified.

**Table 5:** Articular pain change.

First Author, Publication Year	Label	Baseline	12 Weeks	60 Weeks *
Robinson, 2023 [25]	Mean difference from baseline	N/A	−75%	N/S
	Standard deviation	N/A	15.4%	N/S
Connelly, 2020 [26]	Mean value	9	N/S	3
	Standard deviation	2	N/S	3
	Mean difference from baseline	N/A	N/S	6
	Standard error	N/A	N/S	N/S
	95% confidence interval	N/A	N/S	3.6
	95% confidence interval to	N/A	N/S	8.4
	*p* value	N/A	N/S	<0.05

*—mean follow-up time in the study of Connelly et al.; N/A—not applicable; N/S—not specified.

**Table 6:** Mandibular mobility in millimeters.

First Author, Publication Year	Label	Baseline	4 Weeks	12 Weeks	24 Weeks	32 Weeks	48 Weeks	60 Weeks *
He, 2024 [24]	Mean value	13.0	N/S	N/S	N/S	N/S	38.0	N/S
	Mean difference from baseline	N/A	N/S	N/S	N/S	N/S	25.0	N/S
Robinson, 2023 [25]	Mean difference from baseline	N/A	N/S	4.6	N/S	N/S	N/S	N/S
	Standard deviation	N/A	N/S	1.04	N/S	N/S	N/S	N/S
Connelly, 2020	Mean value	31.0	N/S	N/S	N/S	N/S	N/S	36.0
[26]	Standard deviation	5.0	N/S	N/S	N/S	N/S	N/S	5.0
	Mean difference from baseline	N/A	N/S	N/S	N/S	N/S	N/S	5.0
	Standard error	N/A	N/S	N/S	N/S	N/S	N/S	2.4
	95% confidence interval	N/A	N/S	N/S	N/S	N/S	N/S	0.0
	95% confidence interval to	N/A	N/S	N/S	N/S	N/S	N/S	10.0
	*p* value	N/A	N/S	N/S	N/S	N/S	N/S	<0.05
Akhter, 2016 [27]	Mean value	4.8	32.2	33.9	33.9	N/S	33.9	N/S
	Mean difference from baseline	N/A	27.4	29.1	29.1	N/S	29.1	N/S
Bauer, 2013 [28]	Mean value	15	N/S	N/S	N/S	32	N/S	N/S
	Mean difference from baseline	N/A	N/S	N/S	N/S	17	N/S	N/S

*—mean follow-up time in the study of Connelly et al.; N/A—not applicable; N/S—not specified.

**Table 7:** Summary of evidence.

Intervention	Diagnosis	Risk of Bias	Variable	Sample Size	Mean Difference	Standard Deviation	*p* Value
TMJ arthroplasty with interpositional umbilical fat grafting, He, 2024 [24]	TMJ ankylosis	Moderate	Mandibular mobility	1	+192.30%	N/S	N/S
Wharton’s jelly tissue allograft injection into TMJ, Robinson, 2023 [25]	Degenerative joint disease	High	Articular pain	4	−75.00%	15.41%	N/S
			Mandibular mobility	5	N/A	N/A	N/A
Interpositional osteochondral allograft and umbilical cord tissue implantation after TMJ discectomy, Connelly, 2020 [26]	Degenerative joint disease	Moderate	Articular pain	9	−66.67%	29.43%	<0.05
			Mandibular mobility	9	+16.13%	28.65%	<0.05
			Health-related quality of life	9	N/A	N/A	N/A
Application of amniotic membrane over the TMJ condylar process, Akhter, 2016 [27]	TMJ ankylosis	Moderate	Mandibular mobility	13	+452.52%	N/S	N/S
TMJ reconstruction with amniotic membrane after condylar tumor resection, Bauer, 2013 [28]	Giant cell tumor of the mandibular condylar process	Moderate	Mandibular mobility	1	+113.33%	N/S	N/S

N/A—not applicable; N/S—not specified.

## Data Availability

All collected data are included in the content of this article. The protocol was not published prior to the publication of this review. OSF registration number: osf.io/9mnjz.
